# Assessing the effect of BTEX on blood and spirometry parameters staff in a petroleum refinery

**DOI:** 10.3389/fpubh.2022.1037413

**Published:** 2022-11-10

**Authors:** Samad Jalilian, Sima Sabzalipour, Maryam Mohammadi Rouzbahani, Ebrahim Rajabzadeh Ghatrami, Leila Ibrahimy Ghavamabadi

**Affiliations:** ^1^Department of Environment, Ahvaz Branch, Islamic Azad University, Ahvaz, Iran; ^2^Department of Fisheries, Faculty of Marine Natural Resources, Khorramshahr University of Marine Science and Technology, Khorramshahr, Iran

**Keywords:** benzene, toluene, ethyl benzene, xylene, blood, spirometry

## Abstract

This study aimed to investigate the impact of BTEX compound on blood and spirometry parameters of staff in the Abadan petroleum refinery (Iran). In 80 staff was examined in terms of BTEX exposure (40 exposed and 40 non-exposed). In this study, the air sampling was carried out according to the NIOSH 1,501 method and an automated hematology analyzer was used to analyze all blood samples to evaluate blood parameters and using a Micro Direct automated computerized spirometer. Spss20 software was used to interpret the performance. According to the obtained results, total BTEX concentrations with the recommended standard level showed that, toluene, ethylbenzene, and xylenes, concentrations in Abadan Oil Refining Company Workers' breathing zone were lower than the TLV-TWA recommended by ACGIH. However, the average concentration of benzene was higher than the allowable limit. Therefore, in this study the effect of benzene on the blood and respiratory parameters of the workers was evaluated, the comparison of the blood and respiratory parameters between the groups of exposed and unexposed workers did not reveal any statistical difference between the groups (*p* > 0.001). The results showed no statistically significant connection between mean blood and spirometry parameters and benzene exposure. Also, based on results the effect of benzene problems needs to be prevented in employees with adequate engineering and management controls and periodic inspection.

## Introduction

Volatile organic compounds (VOCs), are a group of polluting commodities that have been the subject of intense research in recent decades (1). Within the VOCs, there is a subgroup known as BTEX, which is comprised of benzene alkyl-derivatives (benzene, toluene, ethylbenzene, and xylenes). The monoaromatic hydrocarbons of BTEX cause several negative health effects, including asthma, nausea, dizziness, and inflammation of the nose, mouth, and eyes (2, 3). VOCs are made in liquid or volatile solid form; due to their chemical nature, they evaporate quickly (4, 5). After suspended particles, VOCs are the most abundant and varied emissions (6). BTEX compounds are found abundantly in outdoor air and surface water. They exist in plastic, paint, rubber, detergent, etc., industries as pollutant sources (7, 8). Gas stations are one of the most important sources of emissions of volatile organic compounds and BTEX (9).

Employees are exposed to benzene, toluene, ethylbenzene, and xylenes (10). Several studies have shown that, VOCs in urine, blood and breath tend to be related to corresponding levels in the air levels (11, 12). Long-term exposure to petroleum products has a significant effect on the respiratory systems of some workers (13). Several studies have explored the effects of age, sex, work experience, workplace status, demographic characteristics of individuals, etc., on pulmonary function indices (14). Spirometry is the most important and accessible method for the lung function test. They measured exhalation to first second exhalation and the ratio to assess lung function, critical compression capacity, and maximum volume (13, 14). If spirometry is regular and at intervals specifically performed on workers exposed to respiratory contaminants, it can indicate pulmonary dysfunction before clinical signs and even before the appearance of abnormal findings on the chest (15).

The number of blood cells in healthy people is relatively constant and varies by many factors, including occupational factors (16). Blood cell count (CBC) can be used as an appropriate criterion for diagnosing a variety of blood and non–blood diseases (17). Toxicological studies on humans showed that BTEX compounds are well–absorbed by the body because they are lipophilic in nature and can accumulate in fat-rich tissues such as the brain, bone marrow, and body fat (18). BTEX compounds enter the ambient out door of the city through the exhaust, engine, carburetor of vehicles and also due to the evaporation of gasoline from petroleum products distribution stations, and in this way, the employees of gas stations are exposed to these compounds. The main characteristic of these compounds is their high evaporation rate (19). This study aimed to investigate the impact of the BTEX compound on the blood and spirometric parameters of the personnel of the Abadan oil refinery (Iran) in 2021.

## Materials and methods

**Figure 1:**
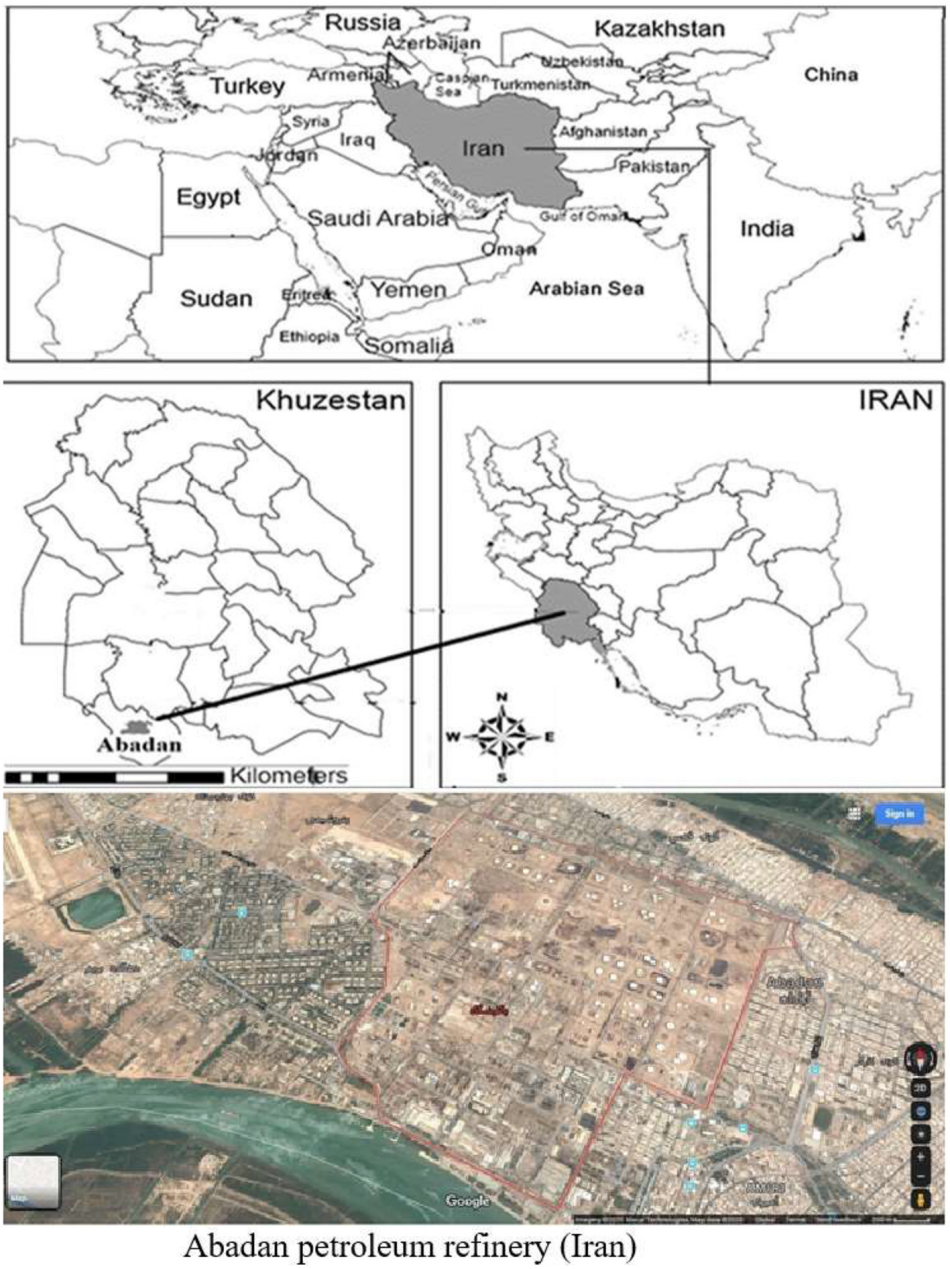
Location of the study area Abadan Oil Refining Company, in the south west of Iran.

Eighty staff was examined in terms of BTEX Exposure (40 exposed and 40 non–exposed). All participants were male and had no history of smoking, Acute illness, systemic disease, or a history of malignancy in the family. The workers' demographic characteristics and general information such as body weight, height, age, and work years were collected using reviewing periodic examinations of workers. Then, informed consent forms were completed for all participants before entering the study. Employees with at least one year of experience were also evaluated. This study was carried out after obtaining permission from the islamic azad university of ahvaz branch and the approval of the management of abadan oil refining company and complying with ethical issues including employee satisfaction (Figure 1). The sample size according to the standard deviation and the allowable error rate in past studies. Also, according to the area, the number of workers and the number of jobs are divided by quota and among the employees working in an 8-h shift, who had the largest number, and among the employees of industrial units (the exposed group who worked in the most critical areas and had had the most work experience) and employees of administrative departments (non-exposed group) and were selected from among matched groups. The demographic characteristics and medical history of the employees were extracted from the electronic file of periodical examinations. Staff members with at least 1 Year of experience were evaluated. Air sampling and BTEX analysis were carried out following NIOSH method number 1,501.

### Sampling and analysis of BTEX

Eighty air samples were taken from 40 exposed workers (two samples per worker). The NIOSH 1,501 method was followed for sampling. Following this method, samples were taken using activated carbon and a low-flow SKC pump (EX 44–224). For sampling and testing, activated SKC tubes were calibrated by pumping a rotary counter (226–01). Samples were collected from the workers' respiratory space for 8 h after preparing the sorbent and sampling pump. This study used an adsorbent every 4 h, set the pump flow to 0.2 mL/min, and used control samples. After collecting the samples using a chemical recycling assay, carbon disulfide solution was used and working standard solution concentrations of 5.1 and 30 μg/ml were tested. The working standard solution was injected into a GC-FID using a 5 ml syringe. The first step was to inject the main sample into the GC-FID. The sample volume was then determined from the standard curve (20).

### Sampling and analysis of blood parameters

From the peripheral vein (median cubital vein), an amount of 3 ml of venous blood was obtained and then immediately transferred to the anticoagulant bottle. An automated hematological analyzer (Sysmex-KX-21N, Japan) was used to analyze all blood samples to assess blood parameters including white blood cells (WBC), hemoglobin (Hb), platelet (PLT), red blood cell (RBC), and hematocrit (Hct) in the laboratory.

### Sampling and analysis of spirometry parameters

The participants were first given an overview of the spirometer's principles, process, and procedure for conducting lung function research (LFT). The spirometer was used to record the age, date of testing, height, name, and weight of the participants. The LFT was performed using a spirometer following the guidelines of the American Thoracic Society. During working hours, LFT was monitored in the workshop office using a Micro Direct automatic computerized spirometer (model MIR010; Italy) (21).

Three spirometry readings were taken from each subject, and the best of the three was chosen. The final reading was based on a variation of 5. Forced vital capacity (FVC), forced expiratory volume in 1 second (FEV1), and FEV_1_ / FVC were all measured using an industry standard automated spirometer. The evaluation in this study was based on the official ATS / ERS technical specifications. According to these specifications, the FVC should be 80% of the expected value for height, weight, age and sex, the FEV_1_ should be 80% of the expected value, and the FEV_1_/FVC ratio should be 70% (22).

### Body mass index

BMI was measured as weight (kg) divided by square of height (in meters) and graded using the international classification scheme of World Health Organization (WHO) as underweight (<18.5 kg/m^2^), normal weight (18.5–24.9 kg/m2), overweight (25–29.9 kg/m^2^), obese (30–39.9 kg/m^2^), and obese class III (herein referred to as “severely obese”) (≥40 kg/m^2^) (23).

### Statistical evaluation

Data analysis SPSS version 20 was used to analyze the obtained data (IBM Corp., Chicago, IL, United Stated). The relationship between physiological variables of exposed and non–exposed employees was investigated using the *t*-test. The correlation between blood and respiratory parameters and demographic characteristics was investigated using the Spearman correlation test. Finally, the correlation between blood and respiratory parameters was investigated using the Pearson coefficient test.

### Ethical approval

Ethics License of the present study was acquired from the Ethics Committee of Ahvaz Branch, Islamic Azad University, Ahvaz, Iran (Ph.D. project with the code 1064817545244271398162301989). According to the national guidelines, studies such as this do not require individual consent.

## Results

This survey was conducted in several units (10 units) of Abadan Oil Refining Company and this survey was conducted with the participation of qualified personnel with experience and familiarity with the daily processes in the operational units of the Abadan Oil Refining Company. Employees had to work 40 h a week. Shift workers were exposed to BTEX compounds 30 times a week, in three half-hour shifts in the morning and three half-hour shifts in the evening, for a total of 3 h in 8 working hours. The average age, years of experience, and BMI (kg/m^2^) of the exposed cases were 37.65 ± 6.65, 9.70 ± 5.74, and 27.47 ± 3.90, respectively. Meanwhile, for those not exposed to BTEX, these values were 38.65 ± 8.53, 8.05 ± 7.22, and 28.9 ± 4.18, respectively.

In this study, worker exposure to BTEX compounds was measured after selecting the relevant occupational exposure groups. According to the results obtained, the total concentrations of BTEX with the recommended standard level, toluene, ethylbenzene and xylenes, showed that the respiratory zone of the workers was lower than the TLV-TWA recommended by ACGIH. However, the average benzene concentration was above the permitted limit (Table 1). Therefore, in this study, the effect of benzene on blood and respiratory parameters of employees was evaluated.

**Table 1:** Mean of BTEX (concentrations in breathing air zone).

**Pollutant**	**TLV-TWA (ppm)**	**Concentrations**
	**in ACGIH**	**(ppm)**
Benzene	0.5	0.7850
Toluene	20	0.4011
Ethylbenzene	20	0.6645
Xylene	100	0.1034

Table 2 Showed that mean blood parameters in the exposed and non-exposed group are in the normal range and Comparing blood parameters between the group of exposed and non-exposed employees revealed no statistically significant difference between the studied groups in terms of the mean blood parameters including white blood cells (WBC), hemoglobin (Hb), platelet (PLT), red blood cell (RBC), and hematocrit (Hct) (*p* > 0.001).

**Table 2:** Relationship between blood and spirometry parameters in workers exposed and not exposed.

**Parameters**	**Exposed (*n* = 40)**	**Non-exposed (*n* = 40)**	**Normal range**	**t**	***P* (0.001)**
WBC (103/ul)	6,967.50 ± 1,209.65	7,647.50 ± 1,699.01	4,000-1,0000	−2.062	0.043
RBC (106/ul)	4.55 ± 0.38	4.88 ± 0.51	4.5-6.3	−3.284	0.002
Hb (g/dl)	13.56 ± 0.86	13.74 ± 1.27	14-167	−0.770	0.444
PLT (103/ul)	257.90 ± 54.79	241.35 ± 55.51	150-450	1.342	0.184
Hct (%)	40.10 ± 2.99	40.10 ± 3.70	40-50	−0.290	0.773
FVC (%)	3.84 ± 0.7305	4.02 ± 0.6024	%80<	−1.197	0.235
FEV_1_ (%)	3.23 ± 0.5319	3.38 ± 0.4673	%80<	−1.418	0.160
FEV_1_/FVC (%)	81.79 ± 9.64	84.65 ± 5.01	%80<	−1.667	0.100

Independent sample t-test; SD, Standard deviation.

Table 3 showed that mean spirometry parameters in the exposed and non-exposed group are in the normal range.

**Table 3:** Results of lung function parameters test in in workers exposed and not exposed.

**Respiratory status**	**Predicted percentage in AST**			**Exposed (%)**			**Non-exposed (%)**		
	**FVC**	**FEV_1_**	**FEV_1_/FVC**	**FVC**	**FEV_1_**	**FEV_1_/FVC**	**FVC**	**FEV_1_**	**FEV_1_/FVC**
Normal	%80<	%80<	%80<	89.42	90.80	80.80	92.35	95.48	90.75
Obstructive	%80<	%80>	%80<	–	–	–	–	–	–
Restrictive	%80>	%80<	%80<	–	–	–	–	–	–
Hybrid	%80>	%80>	%75>	–	–	–	–	–	–

The Spearman correlation test showed that blood and respiratory parameters were not significantly correlated with the demographic characteristics in the studied groups (Table 4) (*p* > 0.001).

**Table 4:** Relationship between physiological parameters and demographic characteristics.

**Parameters**		**Exposed (*****n*** = **40)**			**Non-exposed (*****n*** = **40)**		
		**Age year**	**Experience years**	**BMI**	**Age year**	**Experience years**	**BMI**
WBC	Sig	0.747	0.210	0.327	0.860	0.972	0.882
	R^2^	0.053	0.203	0.159	−0.029	−0.006	0.024
RBC	Sig	0.553	0.0790	0.604	0.990	0.605	0.163
	R^2^	−0.097	−0.281	0.084	0.002	0.084	−0.225
Hb	Sig	0.733	0.424	0.057	0.797	0.814	0.332
	R^2^	−0.056	0.130	−0.303	0.042	0.038	0.157
PLT	Sig	0.989	0.500	0.873	0.846	0.767	0.155
	R^2^	−0.002	0.110	−0.026	−0.032	−0.048	0.229
Hct	Sig	0.149	0.869	0.810	0.478	0.522	0.532
	R^2^	−0.232	0.027	−0.039	−0.116	−0.104	0.102
FVC	Sig	0.390	0.320	0.141	0.142	0.224	0.239
	R_2_	0.140	−0.161	−0.237	−0.237	−0.196	0.190
FEV_1_	Sig	0.313	0.424	0.225	0.153	0.233	0.236
	R^2^	0.164	−0.130	0.198	−0.230	−0.193	0.192
FEV_1_/FVC	Sig	0.052	0.099	0.657	0.347	0.511	0.982
	R^2^	−0.031	0.263	−0.072	0.153	−0.193	0.004

The results of the Pearson statistical test showed significant relationship between hematocrit with red blood cells and Hb in the exposed staff. There is also a significant relationship RBC with FEV_1_/FVC ratio. There was a significant relationship between FVC and FEV_1_, as well as a significant relationship FVC with FEV_1_and HB. Also, the results of the Pearson statistical test showed that in non-exposed employees, the HCT parameter has a significant relationship with HB and FEV_1_. The test results showed that the respiratory parameter of FEV_1_ was significantly related to the parameters of HB, and FVC, and the parameter of FVC was significantly related to the parameters of HCT and FEV_1_/FVC (Table 5).

**Table 5:** Regression of blood and respiratory parameters in the exposed and non—exposed group.

**Parameters**		**Exposed (*****n*** = **40)**								**Non-exposed (*****n*** = **40)**							
		**HCT**	**HB**	**PLT**	**RBC**	**WBC**	**FEV_1_**	**FVC**	**FEV_1_/FVC**	**HCT**	**HB**	**PLT**	**RBC**	**WBC**	**FEV_1_**	**FVC**	**FEV_1_/FVC**
HCT	Sig	1	0.003	0.789	0.877	0.004	0.623	0.320	0.978	1	0.000	0.466	0.159	0.380	0.626	0.507	0.549
	R^2^	1	0.454**	0.044	−0.005	0.445**	0.079	0.161	0.004	1	0.772**	−0.119	0.277	0.143	0.329*	303	0.098
HB	Sig	0.003	1	0.200	0.937	0.189	0.209	0.033	0.361	0.000	1	0.288	0.25	0.771	0.012	0.020	0.570
	R^2^	0.454**	1	0.204	0.014	0.212	0.203	0.337*	0.148	0.772**	1	−0.169	0.184	0.047	0.329*	0.368*	0.092
PLT	Sig	0.789	0.206	1	0.002	0.065	0.137	0.140	0.899	0.466	0.298	1	0.202	0.234	0.807	0.939	0.276
	R^2^	0.044	0.204	1	0.990**	0.995**	−0.239	−0.238	0.021	−0.119	−0.169	1	−0.206	0.193	−0.400	0.013	0.177
RBC	Sig	0.877	0.937	0.990	1	0.972	0.954	0.493	0.045	0.159	0.255	0.202	1	0.109	0.407	0.162	0.480
	R^2^	−0.25	0.014	0.002	1	−0.006	0.009	0.112	0.319*	0.277	0.184	−0.206	1	257	0.135	0.071	0.115
WBC	Sig	0.004	0.189	0.065	0.972	1	0.787	0.379	0.927	0.380	0.777	0.234	0.109	1	0.925	0.866	0.314
	R^2^	0.445**	0.212	0.295	−0.006	1	−0.045	0.143	0.015	0.143	0.047	0.193	0.257	1	0.015	0.028	0.163

The * symbol indicates the correlation significant at the 0.05 level (2-tailed). The ** symbol indicates the correlation significant at the 0.01 level (2-tailed).

## Discussion

The most dangerous BTEX team compounds are benzene (Group 1). Increased risks of leukemia, hematopoietic cancers and respiratory diseases are linked to exposure to these compound (24). Given that, benzene is a harmful pollutant due to its high acute and chronic toxicity. Hence, much effort is needed to measure, control, and lower the negative health impacts of this pollutant. Therefore, control measures should always be taken into consideration.

The results showed, toluene, ethylbenzene, and xylenes, concentrations were lower than the TLV-TWA recommended by ACGIH. Based on the research, oil and gas production workers are exposed to different levels of chemical substances, which generally occur in low concentrations in routine activities. Additional exposures that typically occur in less than a full work shift may occur during maintenance tasks (for example, when containers or tanks require people to enter for cleaning). Also, in operational activities, skin exposures may be high throughout the year, but such exposures have not been investigated on a daily basis. According to the conducted research, Batix compounds have the highest concentration among volatile organic compounds. The results obtained in this study showed that employees were exposed to concentrations of Batix polluting compounds during work. The comparison of the average concentration of Batix compounds with the level of recommended standards showed that the average concentration of toluene, ethylbenzene and xylene compounds in the respiratory zone of the recommended employees is lower than the permissible limit of the OEL standards of Iran and ACGIH of the United States, and this result can be due to the low The concentration of toluene, ethylbenzene and xylene compounds in the raw materials used in operational units, or due to engineering controls and control of possible leaks of these compounds during operation and the presence of proper ventilation in operational units.

Also, Batix compounds are not part of the raw materials used in Abadan Oil Refining Company and the concentration of toluene, ethylbenzene and xylene compounds in raw materials is low, which is one of the important reasons for the low level of these compounds in the breathing area of the employees. It is worth noting that the average concentration of benzene in the respiratory area of the exposed employees was higher than the standard limit, which needs to find its main source and continue the necessary measures to control it until the result is achieved. The reason that BTEX compounds did not affect the lipid parameters of the employee group and there is no significant difference between the parameters of the employee group and the employees of the administrative departments, can be due to the lack of work experience, annual periodical examinations and sufficient rest between work shifts and circulation. The periodic shift and movement of employees (which has caused employees to be exposed to low concentrations in a shorter period of time), the presence of health experts (occupational health, nutrition, physical education) for the necessary controls and training of employees, and the existence of facilities Health care (well–equipped health centers and hospitals) should be suitable for early diagnosis of physiological problems and screening of employees in terms of occupational and non–occupational diseases. Nevertheless, more long-term tests are needed to investigate the exposure to BTEX compounds and volatile organic compounds, and long-term exposure to these compounds, although at a low concentration, has adverse consequences, and it is necessary to use sufficient engineering and management methods and conduct examinations. Periodically, to prevent the adverse health effects of these compounds for employees.

Our results showed, blood and respiratory parameters were not significantly correlated with the demographic characteristics in both groups. On the contrary, several studies have explored the effects of age, sex, work experience, workplace status, demographic characteristics of individuals, etc. on pulmonary function indices (14). The results obtained by comparing blood and respiratory parameters between the group of exposed and non-exposed employees showed that the mean Blood and Spirometry Parameters were not significantly different between the two classes. This means that due to the low concentration of Benzene compound in the respiratory tract of exposed employees, these compounds probably did not affect the blood and respiratory parameters of these employees. Also, the reduction of employees' exposure due to the reduction of shifts and working hours is one of the reasons for the ineffectiveness of Benzene compound on the blood and respiratory parameters of exposed employees. In other study, Tsai et al. (25) compared 1,200 petrochemical staff exposed to varying benzene concentrations with 3,227 non-exposed ones.

They observed no anomalies in the WBC (blood) parameters of white blood cells, red blood cells, and Hb (hemoglobin). Therefore, the results of the above study are consistent with the results of the ongoing study. Pearson's statistical test results showed a significant association between red blood cell hematocrit and Hb in exposed personnel. There is also a significant relationship between RBC and FEV_1_/FVC ratio. There was a significant relationship between FVC and FEV_1_, as well as a significant relationship FVC with FEV_1_ and HB. The results of the Pearson statistical test showed that in non-exposed employees, the HCT parameter has a significant relationship with HB and FEV_1_. The test results showed that the respiratory parameter of FEV_1_ was significantly related to the parameters of HB, and FVC, and the parameter of FVC was significantly related to the parameters of HCT and FEV_1_/FVC (25).

Lad et al. (26) assessed lung function including vital capacity under FVC, FEV_1_, and FEF25-75 pressure values between normal weight and obese subjects. The results showed a significant difference between these two groups. Another study showed that chronic exposure to organic solvents causes an increase in asthma symptoms (27). Murayama et al. (28) examined the absorption of benzene, toluene, and xylene compounds through the human respiratory system using different concentrations in inhalation and exhalation. They reported that the concentration of these BTEX compounds increases in the early stages of exposure but decreases absorption after several hours of exposure. They also stated that measuring the concentration of these compounds in the tail and exhale provides a simple way to estimate the rate of respiratory exposure to these substances.

Other studies do not agree with the findings of this study. For example, after controlling for important confounders, Neghab et al. studied harmful occupational exposure to gasoline and evaluated liver and blood parameters in Iran. In the exposed group, they observed a considerable reduction in the total number of leukocytes relative to the unexposed group (1,347 ± 4.59, <0.001). Moreover, they noticed a significant increase in neutrophil exposure (75.9 ± 3.50). In this study, the difference between the mean blood parameters of the exposed and non-exposed classes was not significant. Also, since the mean blood and spirometry parameters in the two groups are in the normal range, there seems to be no pathological issue presently. Nevertheless, further longitudinal tests are needed for exposure to low concentrations of chemicals (29).

Although BTEX compounds are not among the raw materials used by Abadan Oil Refining Company, their presence can lead to spills from unit equipment. However, the results of this study showed that benzene poses a high risk. It is therefore important to put control measures in place. Workers should be monitored regularly and chemical cartridge respirators should be used to check for hazardous organic substances such as BTEX compounds. In terms of control intervention, the priority should be to minimize exposure by modifying work practices and ventilation systems, as well as by using appropriate protective equipment. According to the EPA, the simplest way to regulate benzene in petroleum products is to eliminate benzene and replace it with a less harmful material.

## Conclusion

The results showed no statistically significant connection between mean blood and spirometry parameters and benzene exposure. However, the effect of BTEX and benzene problems needs to be prevented in employees with adequate engineering and management controls and periodic inspection. In terms of control intervention, the priority must be minimizing the exposure by changing working methods and ventilation systems, as well as using adequate protective equipment. Overall, continuous measurement of BTEX pollutants in the form of measuring harmful factors in the working environment in the refinery, adding tests of red blood cells, white blood cells, hemoglobin, platelets, and hematocrit in periodic occupational medicine examinations for employees who are exposed to BTEXs, Carrying out protective measures against exposure to these compounds, including technical engineering control, management measures, measurement of BTEX compounds in the air of the workplace and intervention strategies, including employee training, rest and adequate sleep, use pollutant distribution models in a research to estimate the most polluted points with BTEX in the refinery area and the possibility of better management, taking exposure control measures, including technical control, engineering, and management measures, and risk reassessment after the interventions necessary to minimize the pollutants are suggested in this regard.

## Data availability statement

The original contributions presented in the study are included in the article/supplementary material, further inquiries can be directed to the correspondingauthor/s.

## Author contributions

SJ, SS, M-MR, E-RG, and L-IG were principal investigators of the study, drafted the manuscript, and performed the statistical analysis. SJ, SS and M-MR were advisors of the study. All authors contributed to the design and data analysis, assisted in the preparation of the final version of the manuscript, and read and approved the final version of the manuscript.

## Funding

This study was extracted from a Ph.D. project with the code 1064817545244271398162301989.

## Conflict of interest

The authors declare that the research was conducted in the absence of any commercial or financial relationships that could be construed as a potential conflict of interest.

## Publisher's note

All claims expressed in this article are solely those of the authors and do not necessarily represent those of their affiliated organizations, or those of the publisher, the editors and the reviewers. Any product that may be evaluated in this article, or claim that may be made by its manufacturer, is not guaranteed or endorsed by the publisher.
